# Multivariate Machine Learning Framework for Predicting Electrical Resistivity of Concrete Using Degree of Saturation and Pore-Structure Parameters

**DOI:** 10.3390/ma19020349

**Published:** 2026-01-15

**Authors:** Youngdae Kim, Seong-Hoon Kee, Cris Edward F. Monjardin, Kevin Paolo V. Robles

**Affiliations:** 1 School of Civil, Environmental and Geological Engineering, Mapua University, Manila 1102, Philippines; ykim@mymail.mapua.edu.ph (Y.K.); cefmonjardin@mapua.edu.ph (C.E.F.M.); 2 Department of ICT integrated Ocean Smart Cities Engineering, Dong-A University, Busan 49315, Republic of Korea; shkee@dau.ac.kr; 3 School of Graduate Studies, Mapua University, Manila 1102, Philippines

**Keywords:** electrical resistivity, concrete, degree of saturation, machine learning, multivariate regression

## Abstract

This study investigates the relationship between apparent electrical resistivity (ER) and key material parameters governing moisture and pore-structure characteristics of concrete. An experimental program was conducted using six concrete mix designs, where ER was continuously measured under controlled wetting and drying cycles to characterize its dependence on the degree of saturation (DS). Results confirmed that ER decreases exponentially with increasing DS across all mixtures, with R^2^ values between 0.896 and 0.997, establishing DS as the dominant factor affecting electrical conduction. To incorporate additional pore-structure parameters, eight input combinations consisting of DS, porosity (P), water–cement ratio (WCR), and compressive strength (f′c) were evaluated using five machine learning models. Gaussian Process Regression and Neural Networks achieved the highest accuracy, particularly when all parameters were included. SHAP analysis revealed that DS accounts for the majority of predictive influence, while porosity and WCR provide secondary but meaningful contributions to ER behavior. Guided by these insights, nonlinear multivariate regression models were formulated, with the exponential model yielding the strongest predictive capability (R^2^ = 0.96). The integrated experimental–computational approach demonstrates that ER is governed by moisture dynamics and pore-structure refinement, offering a physically interpretable and statistically robust framework for nondestructive durability assessment of concrete.

## 1. Introduction

Concrete’s composite nature plays a crucial role in its long-term durability and performance under different environmental exposures [1,2,3,4]. The interaction among its constituents—cement paste, aggregates, water, and chemical admixtures—enables engineers to tailor the material for improved strength, durability, and service life [5,6,7]. For structures exposed to aggressive environments, such as coastal regions, mix design parameters, including water-to-binder ratio and binder composition, are carefully optimized to enhance impermeability and corrosion resistance, ensuring that reinforced concrete meets durability demands in chloride-rich conditions [8,9,10].

Moisture within the pore network significantly affects both the mechanical behavior and durability performance of concrete [11,12,13,14,15]. Higher pore water content can create conditions that make concrete more vulnerable to deterioration, as the pore solution acts as the electrolyte that supports ionic migration—a fundamental component of the electrochemical corrosion process [16,17]. Thus, the degree of saturation is a critical factor when assessing the corrosion susceptibility of reinforced concrete before active corrosion begins. Moreover, deterioration often manifests as internal cracking due to the expansive nature of corrosion products [18,19]. While minor cracking does not necessarily compromise global structural capacity, it can substantially increase permeability, enabling greater ingress of water and harmful substances such as chlorides [20,21,22]. This accelerates steel corrosion and interacts synergistically with other degradation mechanisms, including carbonation and freeze–thaw cycles [23,24].

Tests commonly interpret regions with higher degrees of saturation as zones of increased corrosion risk, particularly once corrosion progresses and rust formation expands within the concrete [25,26,27]. Saturation also strongly influences the response of nondestructive evaluation (NDE) techniques that rely on electrical properties—such as ground-penetrating radar permittivity [28], half-cell potential [29], and electrochemical current density. Consequently, understanding the moisture state is essential for improving the interpretation of NDE data and achieving reliable condition assessment of reinforced concrete exposed to corrosive environments [30,31,32,33].

Among the available NDE techniques, electrical resistivity (ER) is widely used for evaluating concrete durability due to its simplicity, rapid data acquisition, and cost-effectiveness during field inspections [34,35,36,37]. Many agencies incorporate ER measurements into construction quality control and maintenance protocols [38,39]. Previous studies have demonstrated strong correlations between ER and corrosion rate [40,41,42,43,44], as well as chloride diffusivity [45,46,47,48,49]. Fundamentally, ER reflects the combined effects of moisture content and the microstructural characteristics of concrete, making it a valuable indicator of strength and durability [37]. It also represents the ability of the cementitious matrix to resist the flow of ions when subjected to an externally applied electrical current [50,51].

**Figure 1:**
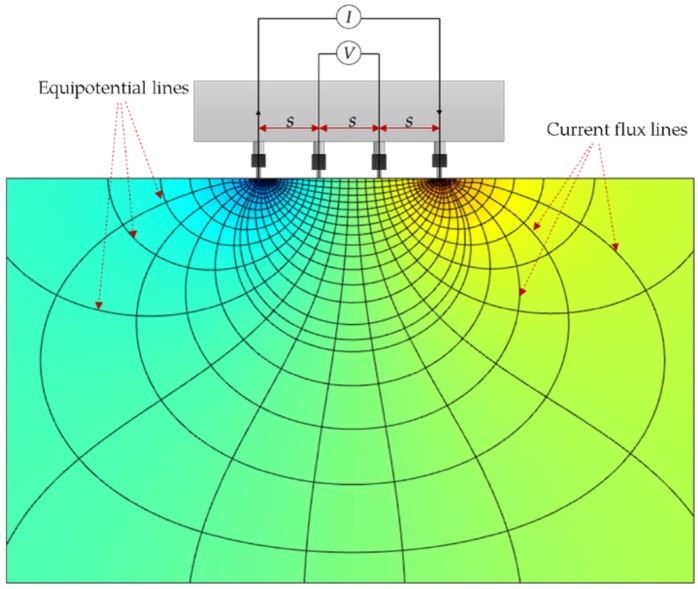
Wenner probe configuration, with ‘s’ as the distance between the electrodes.

Electrical resistivity (ρ) is fundamentally defined as the ratio of the measured electrical potential difference to the applied current, multiplied by an appropriate geometric constant [52]. In the Wenner probe setup, four equally spaced electrodes are arranged linearly, as shown in Figure 1. An alternating current (AC) is applied through the two outer electrodes, while the resulting voltage is captured by the two inner electrodes [53]. The Wenner configuration assumes that the current flows through a semi-infinite, homogeneous, and isotropic medium, an idealization commonly adopted in concrete resistivity measurements [54,55].

Based on these assumptions, the apparent resistivity ($\rho_{app})$ is calculated using Equation (1) where π is the mathematical constant (3.1416), s is the electrode spacing of the Wenner probe (mm), V is the measured electrical potential difference (V), and I is the applied current (A). The value displayed by commercial Wenner devices represents this apparent resistivity and does not explicitly account for the specimen’s thickness or surface area [56]. To obtain the true resistivity (ρ), ($\rho_{app})$ must be corrected using a geometric correction factor, K, as expressed in Equation (2). This factor varies depending on the specimen’s dimensions, surface characteristics, and the specific probe configuration employed [57].

(1) $$\rho_{app}=2\pi s(\frac{V}{I})$$

(2) $$\rho =\frac{\rho_{app}}{K}$$

Accurate interpretation of electrical resistivity (ER) measurements in both laboratory and field settings requires careful consideration of several influencing factors. ER is highly sensitive to material and environmental conditions, and improper handling can lead to misleading assessments. Numerous concrete properties affect ER, including water/cement ratio [58,59], age of concrete [60,61], moisture content and degree of saturation [62], specimen geometry [63,64], temperature [65,66], electrode spacing [67], occurrence of cracks and delamination defects [68,69,70], and presence of re-bars [50,69,71]. Also, the concrete’s microstructure properties, such as the volume and pore size has a direct effect on resistivity measurements [72].

To be specific, a number of studies have observed that the degree of saturation can affect the ER measurements. The increase in water content in concrete significantly decreases ER values [68,73,74,75,76]. Moreover, published papers discussed that for cementitious-based materials, an increase in the degree of water saturation results in a decrease in electrical resistivity [77,78,79,80,81]. To be specific, for concrete specimens, AASHTO TP95-11 indicated that the degree of water saturation has a significant effect on measuring the electrical resistivity of concrete specimens [62]. According to Layssi et al. [72], concrete, being a porous material, which can be related to the water content and degree of pore saturation, may exhibit various conductive and insulating characteristics. For example, electrical resistivity is very high at dry conditions, and the same concrete will be very low in a fully saturated condition [72]. However, to the authors’ knowledge, there is very little (if any) experimental data published regarding the relationship between ER and the degree of water saturation.

Recent studies have increasingly examined the use of regression analysis and machine learning techniques to predict the mechanical properties and performance characteristics of concrete [82,83,84,85]. Regression analysis remains a widely used statistical tool due to its simplicity and computational efficiency, and numerous works have adopted it to estimate compressive strength and related material properties [86,87,88,89]. More advanced data-driven approaches—such as artificial neural networks (ANNs), Gaussian process regression (GPR), and support vector machines (SVMs)—have gained prominence for their ability to model nonlinear and complex relationships directly from experimental data [90,91,92]. ANN models, for example, have been used to predict a variety of concrete mechanical properties [93,94,95] and to enhance the accuracy of testing equipment [96,97]. GPR has likewise shown strong capability in estimating different concrete properties with high precision [98,99,100].

However, to the best of the authors’ knowledge, no existing study has directly correlated the degree of saturation of concrete specimens with nondestructive evaluation parameters such as electrical resistivity. Furthermore, although pore-related characteristics—such as porosity, water–cement ratio, and compressive strength—are known to strongly influence moisture transport and ionic conduction [101,102,103], no published work has simultaneously incorporated these parameters alongside degree of saturation within a unified predictive or analytical framework. This research gap highlights the need for a comprehensive approach that integrates both moisture-state variables and pore-structure characteristics to improve the interpretation of ER measurements and advance durability assessment methodologies for reinforced concrete.

The main objective of this study is to establish a comprehensive and interpretable prediction framework that explains how the degree of saturation and key pore-structure parameters influence the electrical resistivity of concrete. By integrating experimental measurements, machine learning modeling, explainable AI techniques, and nonlinear regression analysis, the study aims to develop reliable predictive tools that support durability assessment and nondestructive evaluation of concrete.

To achieve this objective, the research began with an extensive experimental program designed to characterize the relationship between electrical resistivity and degree of saturation across six concrete mix designs. The laboratory testing provided continuous measurements during controlled wetting and drying cycles, ensuring a dataset that captured both moisture-state effects and material variability. This experimentally generated dataset was then used to develop several machine learning models, where different combinations of degree of saturation, porosity, water–cement ratio, and compressive strength were evaluated to determine their predictive capability. Following the model development stage, SHapley Additive exPlanations (SHAP) analysis, an interpretability method based on cooperative game theory, was used to quantify the contribution of each input parameter, allowing the identification of dominant predictors and the understanding of nonlinear interactions that shape electrical resistivity behavior. Insights from the SHAP interpretation guided the final stage of the study, in which multivariate nonlinear regression models were formulated and compared to determine the most physically consistent and statistically robust equation for predicting resistivity. Together, these tasks form an integrated workflow that links experimental evidence, machine learning prediction, explainable interpretation, and regression modeling to advance the understanding and practical use of electrical resistivity in concrete durability assessment.

## 2. Experimental Investigation

### 2.1. Design and Fabrication of Concrete Specimens

A total of eighteen plain concrete cylinders, each with a height of 200 mm and a diameter of 100 mm, were prepared at Dong-A University in Busan, South Korea (see Figure 1). The specimens were grouped into six design mixes corresponding to nominal strengths of 21, 24, 27, 40, 50, and 70 MPa. The mixture proportions for each design mix are summarized in Table 1, including water content, cement dosage, sand, coarse aggregates, admixture dosage, and total density. After casting, all cylinders were cured under controlled air-dry conditions in the laboratory at a temperature of 20 ± 3 °C until testing.

### 2.2. Degree of Water Saturation of Concrete Specimens

This experiment aimed to evaluate electrical resistivity (ER) at different moisture levels. To ensure a fully controlled saturation process, all specimens were first oven-dried at 80 °C until a constant mass was reached, ensuring complete removal of internal moisture. After oven drying, the cylinders were allowed to cool for 24 h to return to room temperature before the start of saturation measurement.

**Figure 2:**
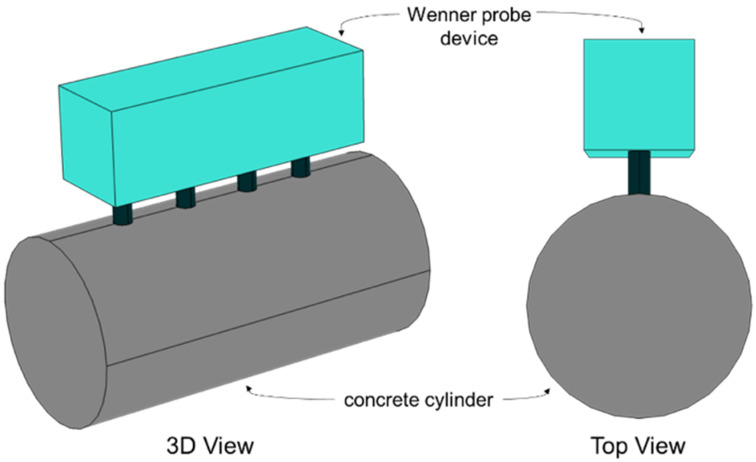
Schematic Diagram of the Test configurations of the ER measurements of concrete cylinders.

From this dry baseline condition, the specimens were fully immersed in water for seven days to achieve complete saturation (Figure 2). The mass of each specimen was recorded prior to immersion and monitored together with ER measurements—every 20 min during the first hour, every 30 min for the next nine hours, and then every 24 h for the remainder of the seven-day period.

Drying was performed in two stages. Initially, the specimens were air-dried at room temperature for seven days. This was followed by oven drying at 80 °C again to reach the oven-dry condition used as the reference mass. The degree of saturation (DS) at any time was calculated as the ratio of the current absorbed water to the maximum water content obtained at full saturation. Equation (3) expresses this relationship, where $m_{i}$ is the mass at a given time, $m_{AD}$ is the oven-dry mass, and $m_{SSD}$ is the saturated-surface-dry mass:

(3) $$DS=\frac{m_{i}-m_{AD}}{m_{SSD}-m_{AD}}$$

### 2.3. ER Measurements of Concrete Cylinders

A commercially available four-point Wenner probe (Resipod, Proceq) with a fixed electrode spacing of 38 mm was used to measure the surface electrical resistivity (ER) of the concrete specimens, as illustrated in Figure 2. The instrument complies with AASHTO T358-15 [104], which specifies procedures for surface resistivity testing as an indicator of concrete’s resistance to chloride ion penetration. During measurement, the device applies a controlled electrical current—ranging from a minimum of 10 μA to a maximum of 200 μA depending on surface contact resistance—and reports the corresponding resistivity in kΩ·cm. This setup was used for all reinforced concrete cubes and plain concrete cylinders included in the study.

For the cylindrical specimens, ER measurements followed the AASHTO TP 95 protocol [65]. As shown in Figure 2, the Wenner probe was positioned parallel to the cylinder height and readings were taken at 90° intervals, completing two full circumferential rotations to capture directional variability. These measurements were performed throughout the saturation process to document changes in apparent resistivity as the degree of saturation increased.

## 3. Machine Learning and Predictive Modelling

**Figure 3:**
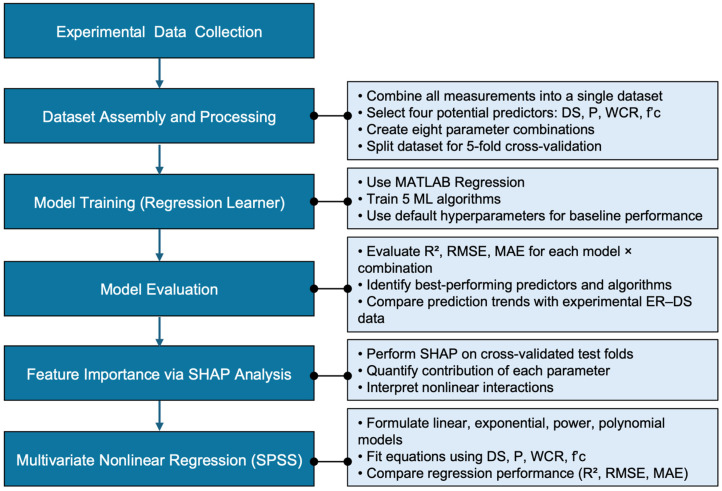
Workflow diagram summarizing the machine-learning and data analysis procedures used in this study.

The machine learning and data analysis component of this study was developed to complement the experimental investigation by establishing a predictive framework capable of capturing the nonlinear relationships governing electrical resistivity. Using the experimentally measured variables—degree of saturation, porosity, water–cement ratio, and compressive strength—the dataset was structured and processed to enable supervised regression modeling. As summarized in Figure 3, the workflow involved preparing the dataset, training multiple algorithms in MATLAB 2025b Regression Learner, evaluating model performance using cross-validated accuracy metrics, and performing SHAP analysis to interpret the relative influence of each parameter. Finally, multivariate nonlinear regression was conducted to derive physically meaningful equations that align with the experimentally observed resistivity behavior. This integrated approach provides both predictive capability and mechanistic insight into moisture- and microstructure-driven resistivity responses in concrete.

### 3.1. Machine Learning-Driven Regression Model

This section presents the development of machine learning regression models used to predict the apparent electrical resistivity (ER) of concrete. The workflow involved dataset preparation, construction of parameter combinations, model training, and performance evaluation to ensure the models generated accurate and reliable predictions.

Four experimentally measured variables were used as potential inputs for the machine learning models: degree of saturation (DS), porosity (P), water–cement ratio (WCR), and compressive strength (f′c). These variables were selected because they represent the primary physical and material characteristics known to influence electrical resistivity in concrete. All measurements used for model development were obtained from the laboratory procedures described in Section 2.

To systematically assess how different combinations of these parameters affect prediction accuracy, eight input-grouping schemes were established, as summarized in Table 2. These combinations range from using DS alone (c1) to expanding the input set to include all parameters (c8). This structured approach allows evaluation of whether incorporating material-related variables improves predictive accuracy beyond saturation alone.

Five machine-learning algorithms were trained using these parameter combinations: Decision Tree (DT), Support Vector Machine (SVM), Boosted Trees (BTs), Gaussian Process Regression (GPR), and Neural Networks (NNs). Each model was tuned and evaluated using standard performance metrics, including R^2^, RMSE, and MAE. Comparing the results across the eight parameter combinations allowed clear identification of which variables contributed to improved predictive accuracy, forming the basis for the model performance analysis presented in Section 4.2.

The five regression models were chosen to capture a broad set of learning strategies, covering linear, nonlinear, and ensemble-based techniques, as outlined in Table 3. These algorithms—commonly applied in previous studies on predicting concrete properties and assessing corrosion—provide an effective compromise between interpretability, model flexibility, and predictive capability [100,105]. Although other techniques, such as Random Forest, Elastic Net, K-Nearest Neighbors, or LightGBM, could also be utilized, the selected group was intended to reflect a representative mix of well-established machine learning approaches suitable for evaluating the proposed hybrid prediction framework [106,107].

The experimental dataset used for training and validating the models consisted of 18 specimens, with each record containing both non-destructive measurement results and corresponding degree of saturation values. Despite its moderate size, the dataset contains sufficient variability for supervised learning tasks, including exploratory regression, feature relevance analysis, and predictive modeling. While machine learning does not require a strict minimum dataset size, prior studies indicate that structured regression analyses can still yield dependable outcomes even with modest sample counts when traditional ML algorithms or shallow neural networks are employed [108,109]. Deep learning methods generally demand substantially larger datasets to avoid overfitting, which is why this study focused on conventional ML algorithms that better align with the scale and characteristics of the available data.

**Table 3:** Summary of the machine learning (ML) algorithms utilized in this study.

Method	Principle	Advantages	Limitations	References
Support Vector Machine (SVM)	Determines the optimal regression or classification boundary by minimizing structural risk.	Flexible control over error tolerance Produces stable solutions through maximization of margin	Difficult to interpret Performance decreases with large or strongly nonlinear datasets unless kernel functions are applied	[110,111,112,113]
Gaussian Process Regression (GPR)	Uses a probabilistic, kernel-based framework to model continuous functions and generate predictions with uncertainty estimates.	Provides confidence bounds for predictions Strong probabilistic foundation enhances reliability assessment	Computationally expensive for large datasets Sensitive to kernel selection and parameter tuning	[114,115,116]
Artificial Neural Network (ANN)	Learns nonlinear relationships by adjusting weighted connections across multiple layers of neurons.	Excellent at representing highly nonlinear patterns Suitable for complex prediction, estimation, and classification tasks	Requires relatively large datasets and higher computational cost Prone to overfitting if not carefully regularized	[117,118,119,120,121]
Decision Tree (DT)	Divides data into smaller subsets through hierarchical feature-based splits to form a tree-like model.	Straightforward to interpret and visualize Works for both regression and classification	Tends to overfit without pruning or additional constraints High variance compared to ensemble models	[122,123,124,125,126]
Bagged Trees (BTs)	Builds multiple decision trees using bootstrap samples and aggregates their outputs to reduce variance.	More robust than a single decision tree Captures complex patterns and reduces overfitting	Computationally heavier than a single tree Reduced model interpretability due to ensemble structure	[127,128]

All models were initially implemented using their default hyperparameter configurations to establish a baseline level of performance. This provides a reference for future work, where dedicated hyperparameter tuning may be conducted to further enhance accuracy and generalizability. The default settings used for each algorithm are listed in Table 4.

To ensure reliable performance assessment, five-fold cross-validation was applied to all supervised learning models. In each fold, 80% of the data was randomly selected for training, while the remaining 20% served as the validation subset. This procedure was repeated five times with different data splits, and the resulting performance measures were averaged across folds to reduce bias and improve robustness. The mean cross-validation error served as the primary criterion for model comparison and selection. This procedure mitigates risks of overfitting and underfitting by evaluating each model on data not used during training within each fold, allowing the researchers to identify the algorithm with the most consistently reliable performance.

### 3.2. Feature Analysis

SHAP analysis was performed to evaluate how each input variable contributed to the predictions of the machine learning models. The computations were carried out in Google Colab using the SHAP library, which provides a model-agnostic approach for interpreting prediction outputs. SHAP, based on cooperative game theory, assigns importance values by estimating each feature’s marginal influence on the model’s prediction. This allows both point-wise interpretation and overall assessment of feature relevance. SHAP values were generated using the test portions of the five-fold cross-validation process, and the mean absolute contributions were normalized such that the total importance for each model summed to 100% [129,130]. This procedure enabled consistent comparison of variable influence across all models and parameter combinations, reflecting both linear and nonlinear relationships captured by the machine learning algorithms.

### 3.3. Multivariable Nonlinear Regression Analysis

A multivariable nonlinear regression analysis was carried out to develop mathematical models describing the relationship between apparent electrical resistivity and the key concrete parameters measured in this study. The analysis was performed using the nonlinear regression module in IBM SPSS Statistics Version 31.0.1.0, which is widely used for fitting complex nonlinear functions to experimental datasets [131]. Several combinations of input variables were tested to evaluate their influence on predicting ER and to determine how moisture-related and microstructural parameters interact within nonlinear formulations.

The SPSS nonlinear regression tool provided flexibility in defining model forms, assigning initial parameter estimates, and iteratively refining the equations until optimal convergence was achieved. This allowed identification of significant predictors and their combined effects, yielding regression equations and corresponding R^2^ values for each parameter set [132,133,134]. The resulting models were compared systematically to determine which combinations produced the best fit to the experimental ER data. This nonlinear regression procedure served as a benchmark for evaluating the performance of the machine learning models presented in later sections, offering insight into the fundamental relationships between degree of saturation, material properties, and electrical resistivity.

### 3.4. Performance Metrics

The performance of each machine learning model was evaluated using standard regression accuracy metrics, including the coefficient of determination (R^2^), root mean square error (RMSE), and mean absolute error (MAE). R^2^ measures how well the model predictions match the experimentally measured electrical resistivity (ER) values. It represents the proportion of the total variability in ER that can be explained by the input variables used in the model. A higher R^2^ value, approaching 1.0, indicates stronger predictive capability and better model alignment with the observed data [135,136]. The metric is computed according to Equation (4), where ${SS}_{\mathrm{residual}}$ is the sum of squared prediction errors and ${SS}_{\mathrm{total}}$ is the total variance of the measured ER values.

(4) $$R^{2}=1-\frac{SS_{\mathrm{residual}}}{SS_{\mathrm{total}}}$$

In addition to R^2^, RMSE and MAE were used to evaluate the magnitude of prediction errors. RMSE quantifies the average size of the error by giving greater weight to larger deviations, making it useful for assessing whether a model produces large outliers in its predictions. MAE, on the other hand, measures the average absolute difference between predicted and observed ER values, providing a straightforward interpretation of typical prediction error. Lower RMSE and MAE values indicate more accurate and reliable model performance. Together, these three metrics allow a comprehensive comparison of the machine learning models, capturing both their overall explanatory power and the precision of their individual predictions.

## 4. Results and Discussion

### 4.1. Relationship Between Degree of Saturation and Apparent Electrical Resistivity

**Figure 4:**
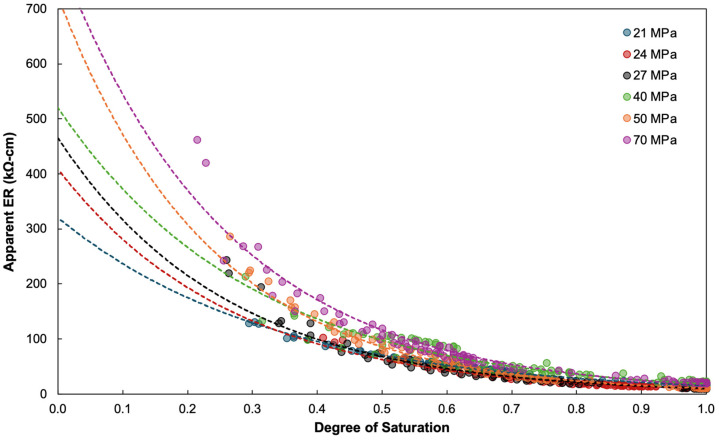
Relationship between the degree of saturation (DS) and apparent electrical resistivity (ER) for concrete mixtures with different design strengths. Dash lines indicate the trend of the experimental data.

The relationship between the degree of saturation (DS) and the apparent electrical resistivity (ER) exhibits a consistent and physically intuitive trend across all concrete mixtures examined. As shown in Figure 4, ER decreases exponentially with increasing DS, regardless of the nominal design strength. At low saturation levels (DS < 0.3), ER values remain high and widely separated among mixtures, reflecting the strong influence of the pore structure during partially saturated conditions. As saturation progresses, the curves converge and ER approaches lower values, illustrating how the presence of interconnected water pathways dominates the conduction mechanism.

Despite the apparent separation of curves by design strength, the behavior is not governed by compressive strength alone. The tabulated regression equations for each mix (Table 5) confirm that the ER–DS relationship is best represented by an exponential decay function. All mixtures exhibit very high goodness-of-fit values, with R^2^ ranging from 0.896 to 0.997, indicating that DS alone captures the dominant mechanism driving electrical resistivity. The 21 MPa mixture, for instance, shows an almost perfect fit (R^2^ = 0.997), while the 50 MPa mixture similarly achieves an excellent R^2^ of 0.984. Even mixtures with greater variability in pore characteristics, such as the 40 MPa and 70 MPa concretes, still maintain high R^2^ values of 0.896 and 0.943, respectively. This consistency reinforces that saturation governs the electrical connectivity of the pore system more strongly than any other individual factor.

However, the separation between the exponential curves corresponding to different design strengths reveals that other material parameters subtly shift the ER response for a given DS. Although DS is measured independently of mechanical properties, it is inherently tied to porosity, which defines the total volume available for water storage [137]. Concrete strength, in turn, is closely linked to water–cement ratio (WCR), a parameter that also influences porosity and pore connectivity [138]. Therefore, mixtures with higher compressive strength generally present lower WCR and denser microstructures, consistent with the higher initial ER values observed at low DS. Conversely, concretes with higher porosity or higher WCR display lower resistivity at comparable saturation levels due to increased ionic mobility within larger or more connected pores [139,140].

These interdependencies demonstrate that while DS directly governs the fraction of conductive pathways filled with water, the characteristics of the pore system—largely shaped by porosity, WCR, and mix strength—modify the magnitude of ER along the exponential decay curve. This explains why mixtures with nominal strengths from 21 MPa to 70 MPa follow the same exponential form but differ in the constants A and B. In this study, the ER–DS relationship for each mixture is described using the general exponential expression $ER=A\;e^{-B\cdot DS}$, where A represents the apparent resistivity under near-dry conditions and B governs the rate at which resistivity decreases as saturation increases. Higher values of A in stronger concretes (e.g., 803.24 for 70 MPa and 721.82 for 50 MPa) reflect their inherently higher resistivity under dry conditions, while wider B values indicate faster resistivity decay as saturation increases. These variations in A and B arise from differences in pore structure, water–cement ratio, and overall microstructural refinement among the mixtures. Ultimately, this behavior confirms that the electrical resistivity of concrete is primarily governed by the combined effects of saturation dynamics and pore structure, rather than compressive strength alone. DS sets the functional shape of the ER response, while microstructural parameters such as porosity and WCR—factors that also influence strength—determine the absolute magnitude of resistivity. The high R^2^ values across all mixtures affirm the reliability of DS as a major predictor of ER, yet the systematic curve separation highlights the need to incorporate additional parameters when developing generalized predictive or machine-learning models, as explored in subsequent sections.

### 4.2. Machine Learning of DS and Apparent ER Including Other Input Parameters

Following the strong inverse relationship established between the degree of saturation and apparent electrical resistivity in Section 3.1, it became evident that ER behavior cannot be attributed to moisture alone. Parameters such as porosity, water–cement ratio, and compressive strength also influence ionic transport and pore solution continuity within concrete, and must therefore be incorporated into predictive models. To examine the combined effects of these variables, eight parameter combinations (c1–c8) were evaluated using five machine learning algorithms: Support Vector Machine (SVM), Decision Tree (DT), Ensemble (BT), Gaussian Process Regression (GPR), and Neural Network (NN). The complete performance metrics (Table 6, Table 7 and Table 8) summarize how accuracy evolves as additional material-related parameters are introduced.

Across all combinations, the GPR and NN consistently outperformed the other models, with the lowest RMSE and MAE values and the highest R^2^ scores. In the baseline case using only degree of saturation (c1), the GPR already achieved relatively high accuracy (RMSE = 15.08 kΩ·cm, R^2^ = 0.91), reinforcing that moisture is the dominant variable affecting resistivity. However, when compressive strength was included (c2), predictive accuracy improved markedly across all models, particularly for GPR and NN, where RMSE dropped to 9.63 and 11.43 kΩ·cm, respectively. This improvement is consistent with the fact that compressive strength indirectly reflects mixture proportioning and pore refinement, influencing the pathways available for ionic movement [141,142].

The addition of water–cement ratio (c3) also enhanced predictive performance, again demonstrating that microstructure-related parameters shape ER behavior. Although c2 and c3 showed similar levels of improvement, combining them (c4) resulted in a slight performance decline for some models, suggesting interaction effects that are better captured when porosity is included. Indeed, when porosity served as a secondary input (c5–c8), model accuracy improved consistently. The effect became most pronounced in combinations c7 and c8, both of which include degree of saturation, porosity, and WCR, with c8 also incorporating compressive strength. Here, GPR yielded exceptional performance, with RMSE values as low as 7.62 kΩ·cm and R^2^ up to 0.98. Neural Networks showed similarly high predictive strength (R^2^ = 0.96 for c7), indicating that datasets incorporating all four parameters best capture the complexity of ER behavior.

While the numerical improvements from c7 to c8 appear modest, the inclusion of compressive strength in c8 remains important. Concrete mixtures with identical porosity and WCR can still differ significantly in microcracking tendency, cement chemistry, and capillary pore refinement—differences that influence electrical pathways. This subtle influence becomes detectable in non-linear machine learning models, particularly GPR and NN, which showed the tightest clustering in the predicted-versus-actual ER plots.

**Figure 5:**
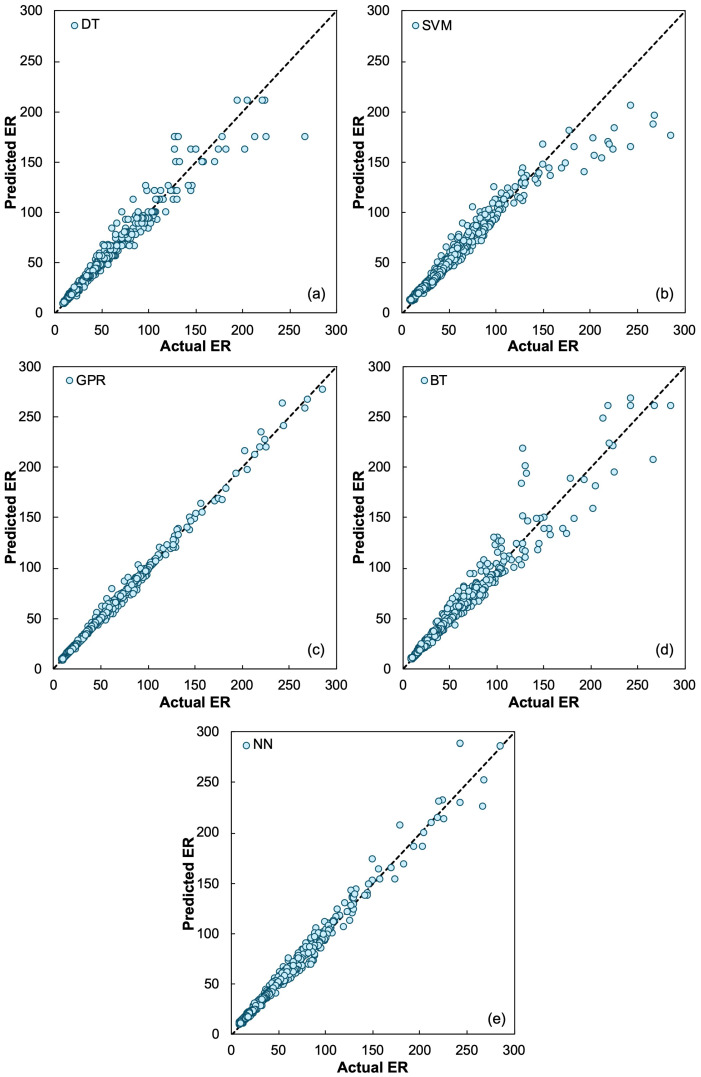
Predicted versus actual apparent electrical resistivity (ER) for the (**a**) Decision Tree (DT), (**b**) Support Vector Machine, (**c**) Gaussian Process Regression, (**d**) Ensemble (BT), and (**e**) Neural Network (NN) models.

The predicted–actual plots (Figure 5) further illustrate these model differences. The Decision Tree model produced a visibly scattered trend, especially at higher ER values, confirming its tendency to oversimplify complex interactions. The Ensemble model demonstrated improved clustering, reflecting reduced variance from tree aggregation. In contrast, the Neural Network displayed strong alignment with the 1:1 line, capturing both the non-linear decrease in ER at low saturation levels and the more gradual changes near full saturation. The GPR model produced similar alignment and was numerically the strongest performer across all parameter groupings.

Overall, the machine learning results confirm that the degree of saturation is the primary driver of electrical resistivity, but its interaction with porosity, WCR, and compressive strength significantly enhances predictive fidelity. Models incorporating all parameters (c8) provide the most balanced representation of concrete’s physical and microstructural behavior, making them valuable tools for forecasting ER-based durability indicators in reinforced concrete.

### 4.3. Feature Contributions and Parameter Interactions Governing Apparent Electrical Resistivity

**Figure 6:**
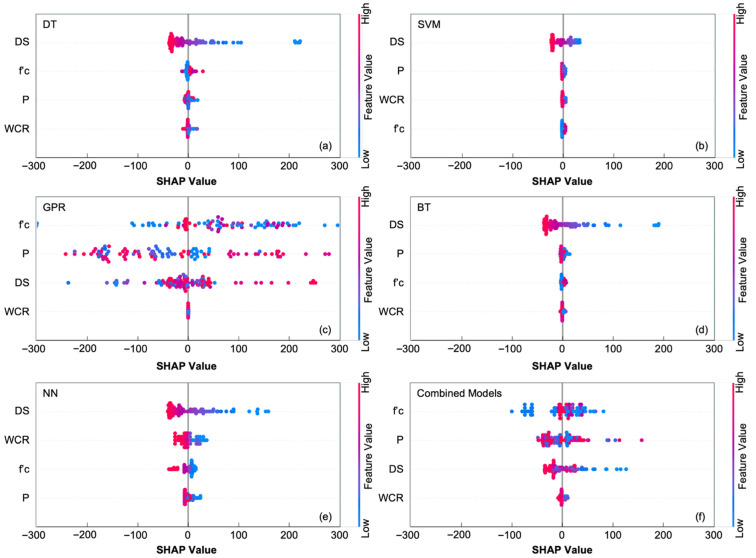
SHAP summary plots showing the distribution of feature contributions for the (**a**) Decision Tree (DT), (**b**) Support Vector Machine (SVM), (**c**) Gaussian Process Regression (GPR), (**d**) Boosted Trees (BTs), (**e**) Neural Network (NN), and (**f**) combined-model evaluation.

The improvements in predictive accuracy observed in Section 3.2 indicate that apparent electrical resistivity cannot be explained by degree of saturation (DS) alone and that microstructural parameters meaningfully influence the response. To understand how each parameter contributes to the predictions, SHAP analysis was performed across all machine learning models. The SHAP summary plots (Figure 6) show that DS consistently dominates model behavior, exhibiting the widest spread of SHAP values and the strongest positive-to-negative gradient. Low DS values push resistivity predictions upward, while high DS values lower them, confirming the exponential decay behavior observed experimentally. This dominance reflects the physical mechanism: moisture saturation controls the continuity of ionic pathways and therefore governs the fundamental shape of the ER response.

Beyond moisture, the SHAP distributions reveal that porosity is the second-most influential parameter, especially in the GPR and NN models, where nonlinear interactions are captured more effectively. Higher porosity values lead to positive SHAP contributions due to increased pore connectivity and easier ion transport, while low porosity reduces predicted ER. The water–cement ratio (WCR) shows a moderate but consistent influence. Although correlated with porosity, WCR retains distinct predictive importance because it affects capillary structure and pore refinement at the material level. Compressive strength, in contrast, exhibits the smallest SHAP variation, indicating that its role is indirect. It serves mainly as a proxy descriptor of mixture proportioning and microstructural density rather than as a direct driver of electrical behavior.

**Figure 7:**
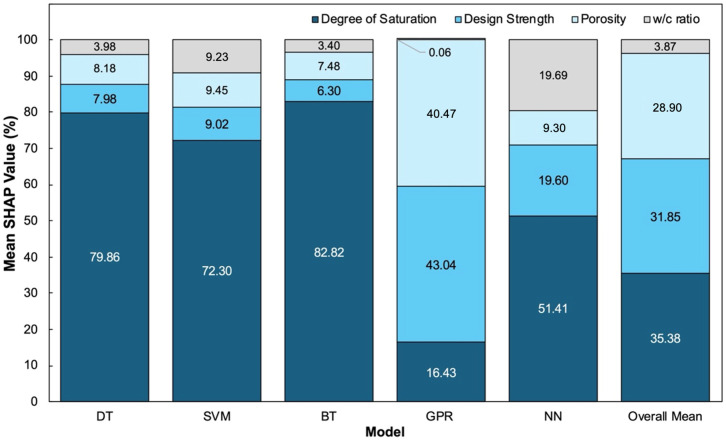
Mean normalized SHAP percentage contributions of each input parameter across all machine learning models.

These trends are further clarified by the mean SHAP percentage values (Figure 7). Across all models, DS accounts for roughly 72–83% of the total predictive contribution, reinforcing its central role in defining ER behavior. Porosity contributes between 9% and 43%, depending on the model’s ability to represent nonlinear effects, while WCR contributes approximately 6–20%, highlighting its secondary but non-negligible role in shaping the magnitude of resistivity. Strength contributes the least (0–20%), consistent with its indirect relationship to conduction pathways.

Together, the SHAP results confirm that ER is governed by the combined effects of moisture dynamics and microstructural refinement. DS dictates the main electrical conduction trend, while porosity and WCR shift the absolute resistivity values by modifying pore volume and connectivity. Strength contributes supplementary information but does not independently control ER. These interpretations explain why models incorporating all four parameters—particularly GPR and NN—demonstrate the highest predictive accuracy and best represent the physical behavior of reinforced concrete under varying saturation conditions.

The predicted–actual plots (Figure 6) further illustrate these model differences. The Decision Tree model produced a visibly scattered trend, especially at higher ER values, confirming its tendency to oversimplify complex interactions. The Ensemble model demonstrated improved clustering, reflecting reduced variance from tree aggregation. In contrast, the Neural Network displayed strong alignment with the 1:1 line, capturing both the non-linear decrease in ER at low saturation levels and the more gradual changes near full saturation. The GPR model produced similar alignment and was numerically the strongest performer across all parameter groupings.

### 4.4. Multivariate Regression Analysis for Developing the Prediction Model

The SHAP results in Section 3.3 demonstrated that degree of saturation (DS), porosity (P), water–cement ratio (WCR), and compressive strength meaningfully influence apparent electrical resistivity (ER). While the machine-learning models revealed how these parameters interact and quantified their relative importance, they do not provide an explicit functional expression suitable for engineering practice. For this reason, the multivariate regression analysis in this section was conducted to translate the ML-derived insights into a transparent and physically interpretable equation. The objective of this section is therefore not to outperform the accuracy of the GPR model, but to develop a closed-form predictive relationship that reflects experimentally observed behavior and can be readily applied in field and design contexts.

Table 9 presents the performance of four regression formulations—linear, exponential, power, and polynomial—constructed using the same input parameters. Among these, the exponential model delivers the strongest predictive capability, achieving the highest R^2^ value (0.96) and lower error metrics relative to the other formulations. This result is highly consistent with the earlier experimental findings in Section 3.1, where the DS–ER relationship for all mixtures was shown to follow a clear exponential decay trend. The strong exponential response observed in both the single-variable experimental analysis and the multivariate context reinforces that ER is fundamentally governed by moisture-driven ionic conduction mechanisms, which naturally exhibit exponential behavior.

The power model also performs relatively well (R^2^ = 0.93), but its higher RMSE and MAE indicate weaker predictive precision. The polynomial regression captures nonlinear interactions through cross-terms and produces acceptable accuracy (R^2^ = 0.90), yet its added complexity may introduce sensitivity or overfitting, especially with limited datasets. In contrast, the linear model performs the poorest (R^2^ = 0.69), reflecting its inability to represent the inherently nonlinear nature of ER. Given these findings, adopting a nonlinear formulation is essential for accurately modeling resistivity behavior.

Overall, the comparison confirms that nonlinear formulations—particularly the exponential model—align most closely with both the experimental behaviour observed in Section 3.1 and the multivariate parameter influences identified through SHAP. Accordingly, the exponential regression equation provides a physically meaningful, statistically robust, and practically interpretable basis for ER prediction, complementing the exploratory insights obtained from the machine-learning analysis for predicting apparent electrical resistivity (ER) using degree of saturation (DS), porosity (P), water–cement ratio (WCR), and compressive strength (f′c).

### 4.5. Summary, Practical Implications, and Recommendations

This study established that the degree of saturation (DS) is the strongest predictor of apparent electrical resistivity (ER), governing the dominant trend of its exponential decay. Machine learning models demonstrated that ER responds to the nonlinear interactions among DS, porosity, water–cement ratio, and compressive strength, with GPR and Neural Networks providing the highest predictive accuracy. SHAP analysis supported these results by illustrating that moisture state drives the primary variability, while microstructural parameters refine the absolute magnitude of ER. The final multivariate regression model—particularly the exponential formulation—proved both accurate and physically consistent with the mechanisms observed experimentally and computationally.

The findings highlight that electrical resistivity is a robust non-destructive indicator of moisture-related deterioration in concrete. Because DS directly controls ionic conduction pathways, ER provides meaningful insight into internal moisture distribution and its impact on corrosion susceptibility. Porosity further influences resistivity by shaping pore connectivity, suggesting that these two parameters should be prioritized during field evaluations. The developed regression model offers a practical tool that can be incorporated into durability assessment workflows, allowing engineers to estimate ER using measurable properties and improve maintenance and monitoring decisions.

Future work should validate the proposed model using larger datasets, additional concrete mixtures, and in-service field measurements to ensure broader applicability. Expanding the dataset is essential because concrete behavior varies significantly with mixture proportions, curing conditions, and environmental exposure; larger and more diverse datasets have been shown to improve model generalization and reduce overfitting in concrete durability prediction studies [143,144]. This comprehensive approach not only enhances predictive accuracy but also supports the development of more effective strategies for assessing concrete durability in varying environmental conditions.

Simulation of saturation processes—particularly moisture transport under changing boundary conditions—should also be explored to better understand how DS evolves within real structures. Prior studies emphasize that moisture movement in concrete is governed by complex diffusion–permeation mechanisms, and numerical simulations provide valuable insight into internal saturation gradients that are difficult to measure experimentally [145]. Such simulations can help interpret resistivity variations more accurately and support the development of moisture-compensated NDT models.

It is also recommended to evaluate different specimen shapes and sizes, as saturation profiles, pore connectivity, and drying–wetting behavior can vary substantially with geometry and volume. Researchers have shown that specimen geometry influences moisture transport rates and can lead to size-dependent differences in measured resistivity, making it important to confirm that predictive models remain reliable across varying dimensions [76,146].

In addition, integrating temperature corrections into ER prediction is necessary because electrical resistivity is highly temperature-dependent; even small fluctuations can significantly alter ionic mobility in the pore solution. Temperature-adjusted resistivity models have been widely recommended to improve accuracy in both field and laboratory applications [147,148].

Finally, combining ER with complementary durability metrics such as half-cell potential (HCP), electrochemical impedance spectroscopy (EIS), or concrete cover depth can enhance corrosion monitoring. Multi-parameter approaches have been shown to improve diagnostic reliability by capturing different aspects of reinforcement corrosion, including electrochemical activity, moisture influence, and transport properties [149,150]. Integrating these methods can therefore lead to more robust and comprehensive assessment frameworks for reinforced concrete structures.

## 5. Conclusions

This study presents an integrated experimental and computational framework that establishes how electrical resistivity in concrete is governed by moisture dynamics and pore-structure characteristics. By combining controlled saturation testing, machine learning prediction, SHAP-based interpretability, and multivariate nonlinear regression, the work provides a physically consistent and statistically robust approach for estimating resistivity across different concrete mixtures and moisture conditions. Based on the results and discussion, the following specific conclusions can be drawn:

1. The degree of saturation was experimentally confirmed as the dominant factor influencing electrical resistivity. Across all six concrete mixtures, ER exhibited a consistent exponential decay with increasing DS, with R^2^ values ranging from 0.896 to 0.997. This demonstrates that the moisture state governs the continuity of ionic pathways and defines the fundamental ER response regardless of compressive strength.
2. Machine learning models revealed that incorporating pore-structure parameters significantly improves ER prediction. Although DS alone provided a strong baseline accuracy, adding porosity, WCR, and compressive strength enhanced model performance. Gaussian Process Regression and Neural Networks consistently achieved the highest accuracy, especially for combinations that included all parameters.
3. SHAP analysis showed that DS accounts for the majority of predictive influence, while porosity and WCR serve as key modifiers of the ER magnitude. SHAP values indicated that DS contributes approximately 72–83% of total importance. Porosity and WCR further refine ER predictions by shaping pore connectivity and microstructural density, whereas compressive strength had a minimal direct effect.
4. Nonlinear multivariate regression confirmed the exponential model as the most physically meaningful and statistically robust equation. Among linear, polynomial, power, and exponential forms, the exponential model achieved the highest R^2^ (0.96). This aligns with the experimentally observed DS–ER relationship and the nonlinear interactions revealed by ML and SHAP.
5. Electrical resistivity demonstrates strong potential as a nondestructive indicator for moisture-related durability evaluation. The results highlight the importance of considering DS and pore-structure characteristics when interpreting ER measurements. The developed regression model can support field assessments, durability monitoring, and integration into ER-based diagnostic tools.

Overall, the study emphasizes that accurate interpretation of ER requires accounting for both the moisture condition and the underlying pore structure. Future work should include larger datasets, different specimen geometries, saturation simulations, temperature correction, and integration with other NDT methods such as HCP or EIS to further enhance predictive capability.

## Figures and Tables

**Table 1:** Concrete mixture proportions for each mix design used in specimen fabrication.

Design Mix (MPa)	Density (kg/m^3^)					
	Water	Cement	Sand	Aggregates	Admixtures	Total
21	168	312	908	931	2.18	2321.18
24	170	330	884	931	1.98	2316.98
27	170	367	858	923	2.57	2320.57
40	163	460	859	887	4.60	2373.60
50	163	529	799	855	5.82	2351.82
70	160	625	725	876	6.88	2392.88

**Table 2:** Parameter combinations used for machine-learning model development.

Combination	Parameters
c1	DS
c2	DS + f′c
c3	DS + WCR
c4	DS + f′c + WCR
c5	DS + P
c6	DS + P + f′c
c7	DS + P + WCR
c8	DS + P + WCR + f′c

**Table 4:** Default hyperparameter settings in MATLAB Regression Learner.

Model Type	Default Hyperparameters
SVM	Kernel: Medium Gaussian Box constraint: 1; Kernel scale: auto Standardize data: Yes
GPR	Kernel: Squared Exponential Basis function: Constant Sigma (noise level): auto-estimated
ANN	Hidden layers: 1 Neurons: 25 Activation function: ReLU Solver: Adam Standardize data: Yes
DT	Minimum leaf size: 4; Split criterion: Mean Squared Error (MSE)
BT	Method: Bag; Number of learning cycles: 30 Minimum leaf size: 8

**Table 5:** Regression equations describing the exponential relationship between apparent electrical resistivity (ER) and degree of saturation (DS) for each concrete mix.

Design Mix (MPa)	Regression Equation	R^2^
21	$ER={320.38e}^{-3.023DS}$	0.997
24	$ER={408.64e}^{-3.744DS}$	0.985
27	$ER={464.88e}^{-3.847DS}$	0.952
40	$ER={520.46e}^{-3.343DS}$	0.896
50	$ER={721.82e}^{-4.259DS}$	0.984
70	$ER={803.24e}^{-3.853DS}$	0.943

**Table 6:** Root Mean Square Error (RMSE) of the five machine learning models using parameter combinations c1–c8.

Combination	RMSE				
	DT	SVM	BT	GPR	NN
c1	22.09	20.37	20.86	15.08	15.21
c2	18.17	21.48	17.50	9.63	11.43
c3	18.18	20.56	17.36	9.72	11.00
c4	18.18	20.28	19.40	13.53	14.16
c5	20.52	22.43	19.91	15.77	16.19
c6	20.80	20.59	19.81	9.43	12.39
c7	18.07	19.05	17.80	8.97	9.92
c8	20.82	19.30	19.02	7.62	12.37

**Table 7:** Coefficient of determination (R^2^) for each machine learning model across parameter combinations c1–c8.

Combination	R^2^				
	DT	SVM	BT	GPR	NN
c1	0.82	0.84	0.84	0.91	0.91
c2	0.87	0.83	0.88	0.96	0.95
c3	0.87	0.84	0.89	0.96	0.95
c4	0.87	0.84	0.86	0.93	0.92
c5	0.84	0.81	0.85	0.91	0.9
c6	0.84	0.84	0.85	0.97	0.94
c7	0.88	0.86	0.88	0.98	0.96
c8	0.84	0.86	0.86	0.98	0.94

**Table 8:** Mean Absolute Error (MAE) values for the machine learning models using parameter combinations c1–c8.

Combination	MAE				
	DT	SVM	BT	GPR	NN
c1	12.15	10.68	11.07	10.12	10.15
c2	8.12	7.79	7.11	4.68	5.54
c3	8.22	7.77	7.45	5.14	5.98
c4	8.22	7.24	7.49	4.95	5.34
c5	7.72	7.76	7.26	6.10	6.85
c6	7.91	6.72	7.05	2.66	4.57
c7	7.35	6.34	6.86	2.58	4.41
c8	7.74	10.68	11.07	10.12	10.15

**Table 9:** Performance of multivariate regression models for predicting apparent electrical resistivity (ER) using degree of saturation (DS), porosity (P), water–cement ratio (WCR), and compressive strength (f′c).

Model	Equation	R^2^	RMSE	MAE
Linear	$ER=353.194-195.489DS-4.685P-195.280WCR-1.169f^{\prime }c$	0.69	28.56	17.98
Exponential	$ER={593.227e}^{\left(-3.707DS-0.078P-0.356WCR-0.006f^{\prime }c\right)}$	0.96	19.39	8.91
Power	$ER=e^{3.786}\cdot {DS}^{-2.378}\cdot P^{-0.699}\cdot {WCR}^{-0.094}\cdot {f^{\prime }c}^{0.417}$	0.93	26.19	10.09
Polynomial	$ER=505.044{DS}^{2}-17.427P^{2}+10.914\left(DS\cdot P\right)+1337.781\left(DS\cdot WCR\right)$ $+8.254\left(DS\cdot f^{\prime }c\right)-613.764\left(P\cdot WCR\right)-1874.381DS+739.750P$ $+33,551.828WCR+248.048f^{\prime }c-13,564.806$	0.90	15.85	9.45

## Data Availability

The original contributions presented in this study are included in the article. Further inquiries can be directed to the corresponding author.
